# CT-based quantification of intratumoral heterogeneity for predicting distant metastasis in retroperitoneal sarcoma

**DOI:** 10.1186/s13244-025-01977-9

**Published:** 2025-05-09

**Authors:** Jun Xu, Jian-guo Miao, Chen-xi Wang, Yu-peng Zhu, Ke Liu, Si-yuan Qin, Hai-song Chen, Ning Lang

**Affiliations:** 1 Department of Radiology, Peking University Third Hospital, No. 49, North Garden Road, Haidian District, Beijing, China; 2 The College of Computer Science & Technology, Qingdao University, No. 308, Ning Xia Road, Shinan District, Qingdao, Shandong China; 3 Department of Radiology, The Affiliated Hospital of Qingdao University, No. 16, Jiangsu Road, Shinan District, Qingdao, Shandong China

**Keywords:** Retroperitoneal tumor, Intratumoral heterogeneity, Distant metastasis, Deep learning

## Abstract

**Objectives:**

Retroperitoneal sarcoma (RPS) is highly heterogeneous, leading to different risks of distant metastasis (DM) among patients with the same clinical stage. This study aims to develop a quantitative method for assessing intratumoral heterogeneity (ITH) using preoperative contrast-enhanced CT (CECT) scans and evaluate its ability to predict DM risk.

**Methods:**

We conducted a retrospective analysis of 274 PRS patients who underwent complete surgical resection and were monitored for ≥ 36 months at two centers. Conventional radiomics (C-radiomics), ITH radiomics, and deep-learning (DL) features were extracted from the preoperative CECT scans and developed single-modality models. Clinical indicators and high-throughput CECT features were integrated to develop a combined model for predicting DM. The performance of the models was evaluated by measuring the receiver operating characteristic curve and Harrell’s concordance index (C-index). Distant metastasis-free survival (DMFS) was also predicted to further assess survival benefits.

**Results:**

The ITH model demonstrated satisfactory predictive capability for DM in internal and external validation cohorts (AUC: 0.735, 0.765; C-index: 0.691, 0.729). The combined model that combined clinicoradiological variables, ITH-score, and DL-score achieved the best predictive performance in internal and external validation cohorts (AUC: 0.864, 0.801; C-index: 0.770, 0.752), successfully stratified patients into high- and low-risk groups for DM (*p* < 0.05).

**Conclusions:**

The combined model demonstrated promising potential for accurately predicting the DM risk and stratifying the DMFS risk in RPS patients undergoing complete surgical resection, providing a valuable tool for guiding treatment decisions and follow-up strategies.

**Critical relevance statement:**

The intratumoral heterogeneity analysis facilitates the identification of high-risk retroperitoneal sarcoma patients prone to distant metastasis and poor prognoses, enabling the selection of candidates for more aggressive surgical and post-surgical interventions.

**Key Points:**

- Preoperative identification of retroperitoneal sarcoma (RPS) with a high potential for distant metastasis (DM) is crucial for targeted interventional strategies.
- Quantitative assessment of intratumoral heterogeneity achieved reasonable performance for predicting DM.
- The integrated model combining clinicoradiological variables, ITH radiomics, and deep-learning features effectively predicted distant metastasis-free survival.

**Graphical Abstract:**

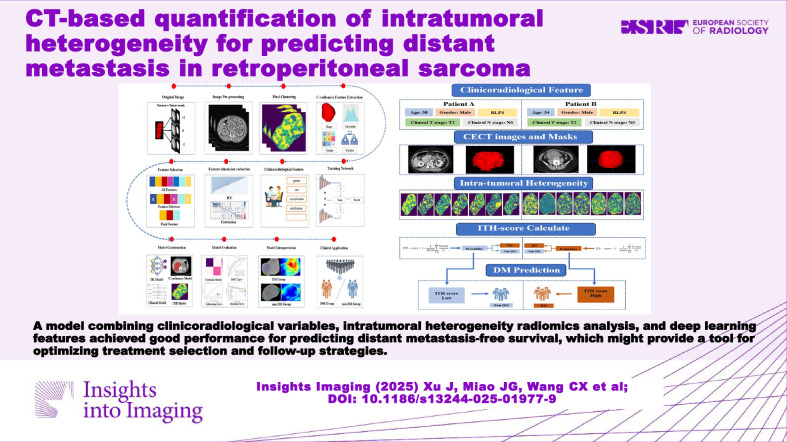

## Introduction

Retroperitoneal sarcomas (RPS) demonstrate significant intratumoral heterogeneity (ITH) and have a poorer prognosis than extremity sarcomas [1, 2]. Liposarcomas and leiomyosarcomas are the predominant histological subtypes of RPS [3, 4]. Although radical resection is the standard curative therapy for localized RPS, 30%–50% of patients still experience distant metastasis (DM) within five years despite undergoing microscopically negative margin (R0) resection [5–7]. Metastatic RPS is linked to an unfavorable prognosis, exhibiting a median survival time of only 16 months and a cumulative 5-year survival fraction below 50% [8]. Neoadjuvant radiotherapy has been definitively shown to enhance the probability of accomplishing complete resection and reduce the risk of peritoneal metastasis [9–11]. However, the potential toxicity of radiotherapy and its effect on patients’ quality of life remain topics of debate [9]. While conclusive evidence supporting neoadjuvant chemotherapy remains controversial, the RPS treatment paradigm is progressively emphasizing personalized strategies and earlier interventions. This shift is epitomized by the ongoing phase III trial NCT04031676 evaluating the efficacy of neoadjuvant chemotherapy (primary completion 2027) [12]. Consequently, preoperative identification of RPS with the potential for DM is crucial for guiding targeted interventional strategies, improving survival, and enhancing quality of life.

The National Comprehensive Cancer Network (NCCN) Guidelines advocate for the use of contrast-enhanced computed tomography (CECT) to track disease progression in individuals with RPS [13]. CECT provides a comprehensive view of tumor tissue by capturing both morphological and texture information [14]. Genomic heterogeneity within the tumor can lead to variations in cellular composition, which may manifest as local or global differences in images. However, conventional CECT interpretation primarily focuses on macroscopic morphology, limiting its ability to identify higher-dimensional characteristics relevant to DM, which are imperceptible to the human eye [15]. Additionally, this approach relies heavily on the subjective judgment and experience of radiologists, leading to potential inter-physician diagnostic variability [16]. Radiomics and deep-learning (DL) techniques can autonomously learn representative information from CECT images, incrementally harvesting features without the need for human intervention. These methods have demonstrated enhanced diagnostic capability and offer supplementary insights for devising subsequent treatment plans [17–19]. For example, Arthur et al [20] developed a radiomics model that could effectively predict the histological type and grade of RPS with outstanding performance. Tian et al [18] applied a ResNet50-based method to extract representative features from CECT images to predict DM of retroperitoneal leiomyosarcomas (RLMS). Despite these significant advancements, previous studies have predominantly focused on the overall features of RPS without delving into the predictive significance of heterogeneity within RPS for the risk of DM.

ITH poses challenges in clinical treatment choices and follow-up management [21], and it has been recognized to play a role in tumor metastasis, progression, and unfavorable clinical outcomes [22–24]. Previous studies have exhibited promising results in the application of ITH quantitative analysis for predicting pathologic complete response and DM in breast cancer [25, 26], as well as the recurrence of hepatocellular carcinoma following liver transplantation [27]. The inherent heterogeneity within the tumor may help explain variations in clinical outcomes among patients with similar clinicopathologic characteristics. Thus, a quantitative measure of ITH could be a valuable biomarker for predicting DM among patients with RPS.

The objectives of this study were threefold. First, we aimed to establish a quantitative assessment of ITH on preoperative CECT images. Second, we conducted ablation and comparative experiments to investigate the predictive efficacy and generalization capabilities of clinical variables, conventional radiomics (hereafter, C-radiomics), ITH, and DL models. Finally, we intended to construct an optimal combined model and evaluate its effectiveness for predicting the distant metastasis-free survival (DMFS) in patients with RLMS and retroperitoneal liposarcoma (RLPS) following complete surgical resection.

## Materials and methods

### Ethics

This investigation adhered to the ethical principles outlined in the Declaration of Helsinki and was granted ethical clearance by the institutional review boards of Peking University Third Hospital (M2023745). Given the retrospective design of the research, the requirement for collecting written informed consent was waived. The present research conformed to the STARD-2015 guidelines [28].

### Participants

**Fig. 1:**
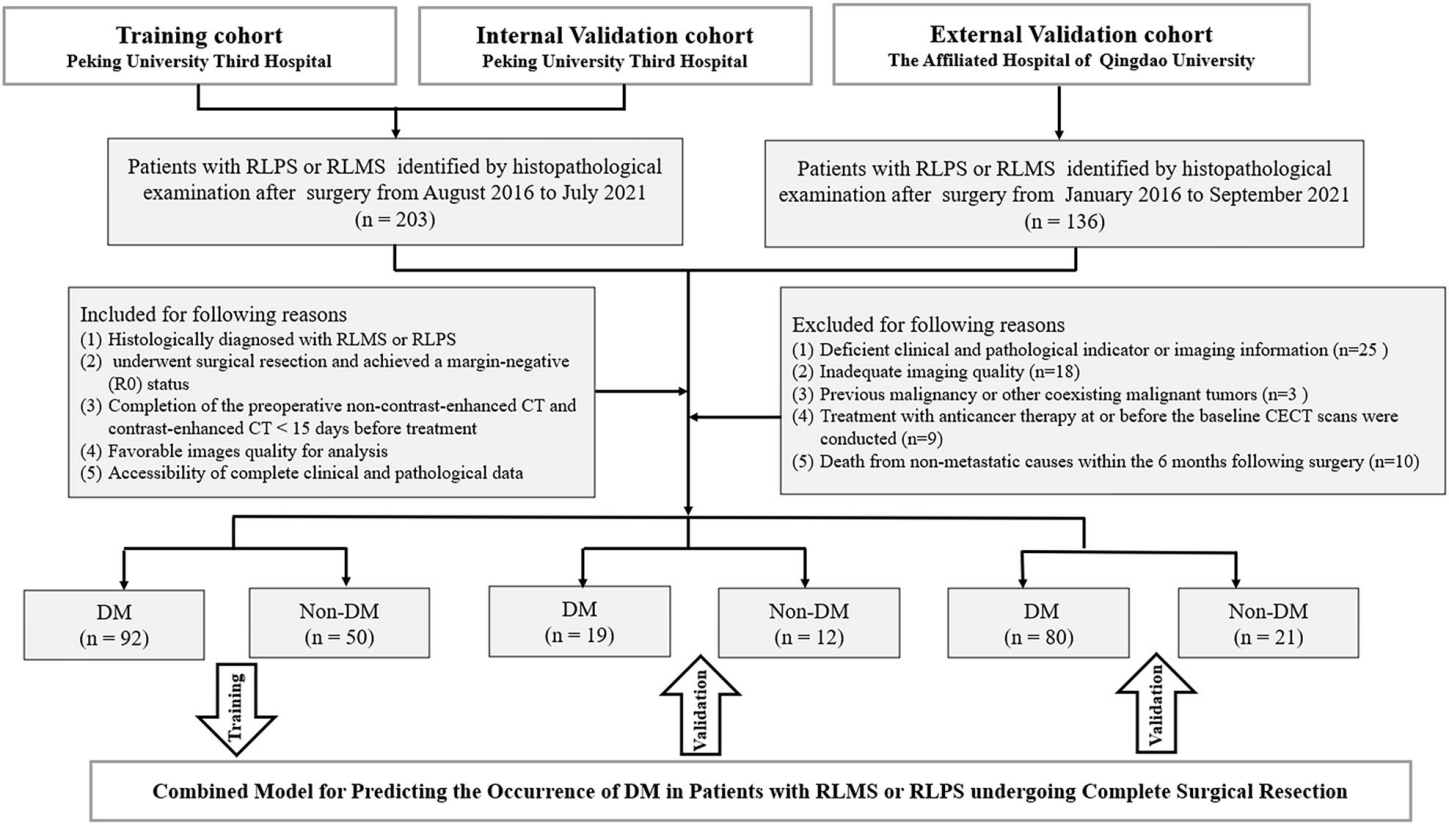
Detailed flowchart of study enrollment

Consecutive RPS patients from two centers who underwent complete surgical resection from January 2016 to September 2021 were retrospectively included, and their CECT images and clinical information were collected. Patients treated at Peking University Third Hospital were randomly allocated to training and internal validation sets in a 4:1 ratio. Patients treated at the Affiliated Hospital of Qingdao University were assigned to an external validation set. The patient selection is depicted in Fig. 1. The detailed inclusion and exclusion criteria are included in Appendix 1.

### Data collection

The demographic data for age and sex were documented for all patients. Three experienced radiologists in abdominal imaging diagnosis independently assessed the following semantic features of CECT scans: tumor number, shape, presence of cystic spaces or necrosis, enhancement pattern, degree of enhancement, adipose tissue, calcification, adjacent tissue involvement, and the type of adjacent tissue involvement (detailed in Appendix 2). The clinical tumor, node, metastasis (TNM) staging was aligned with the 8th edition of the American Joint Committee on Cancer (AJCC) Staging System. Histologic type and grade according to the Federation Nationale des Centers de Lutte Contre le Cancer (FNCLCC) were obtained from the surgical specimen. Histologic types were grouped as follows: well-differentiated liposarcoma (WDLPS), dedifferentiated liposarcoma (DDLPS), other LPS, and leiomyosarcoma (LMS).

### Follow-up assessments

DMFS was defined as the period from surgery to DM (determined by imaging or histopathological confirmation), the final follow-up, or death without disease progression. For the first two years following surgery, patients underwent follow-up evaluations every 3–6 months, after which they underwent bi-annual evaluations. Data tracking was performed through examination of medical records, imaging data, and telephonic consultations. The follow-up cutoff date was March 31, 2024.

### Image acquisition and region of interest segmentation

CECT images in the venous phase are captured between 60 to 70s following injection of non-ionic contrast medium. The administered dose and infusion rate were adjusted based on the patient’s height and weight. The acquisition parameters for CECT are shown in Table S1. Detailed segmentation methodology, including preprocessing steps and interobserver review, is described in the Appendix 3, 4 and Fig. S1.

### C-radiomics analysis

Pyradiomics (version 3.1; https://pyradiomics.readthedocs.io) was employed to extract 1321 radiomic features from the whole ROIs. The Combat Harmonization method employs the use of different scanner models in batches to ensure consistency and reliability in image analysis (detailed in Appendix 5) [29]. Next, C-radiomics features with high stability, as determined by intraclass correlation coefficient (ICC) > 0.90, were preserved. To further refine the features, the minimum Redundancy Maximum Relevance (mRMR) algorithm and Least Absolute Shrinkage and Selection Operator (Lasso) regression were employed. The C-radiomics model was finally developed with 20 features. We employed the Random Forest classifier to devise a predictive model, augmented it with the Synthetic Minority Oversampling Technique (SMOTE) to mitigate the issue of imbalanced data, and leveraged Bayesian hyperparameter optimization along with ten-fold cross-validation to achieve automatic model tuning.

### ITH radiomics analysis

The ITH-score, a comprehensive metric that integrates local and global radiomics data to quantify intra-tumor heterogeneity, has been described previously [30]. An outline of the process for calculating this score is provided in the following sentences. First, 104 C-radiomics features were extracted from each pixel within the tumor region to characterize the local information using PyRadiomics. Second, we clustered pixels based on their local information, with pixels sharing the same cluster label exhibiting comparable intensity and neighboring texture. ITH-score computation based on subcluster spatial characteristics. Key spatial parameters were defined as follows: The number of connected regions within cluster $i$ is denoted by ${n}_{i}$, and ${S}_{i,\max }$ represents the largest area among these regions. $V$ indicates the total number of clusters, while ${S}_{{total}}$ indicates the entire tumor area. Subsequently, we used the following formula to quantify tumor region heterogeneity:

$${{\mathrm{I}}}{{{\mathrm{TH}}}}-{{{\mathrm{score}}}}=1-\frac{1}{{S}_{{{{total}}}}}{\sum }_{i=1}^{V}\frac{{S}_{i,\max }}{{n}_{i}}$$

The ITH-score ranges from 0 to 1, with higher scores indicating greater dissemination in the label map, which reflects increased heterogeneity in both cellular composition and spatial distribution within the tissue. We evaluated the performance of the ITH-scores obtained from 2 to 8 clustering configurations (detailed in Appendix 6 and Table S2). To further enhance the stability, we integrated ITH-scores derived from 2 to 5 clusters and collectively input them into the RF model. The resultant composite model outperformed all single-cluster-number models, suggesting that the integrated approach significantly enhances the robustness of ITH analysis. Additionally, we assigned colors to pixels on the basis of cluster labels, thereby creating a label map that visually represents the global distribution and facilitates quantification of ITH (Fig. S2).

### DL model development

Our study implemented a 2.5D-CNN architecture, which was trained on CECT images, to forecast the postoperative DM risk in patients with RPS. Two distinct DL approaches were employed for model construction: feature extraction DL (DL_F) model and end-to-end DL (DL_E) model. DL models typically require large datasets for training, making data augmentation essential to prevent overfitting and improve model robustness [31]. In this study, we applied flipping and rotation as augmentation techniques to all images in the training set [32]. The flipping operation included horizontal flipping, while rotation involved increments of 45° from 0° to 360°, specifically at 45°, 90°, 135°, and so on. These augmentations did not alter the tumor characteristics. To further elevate model performance, we developed a CNN that integrated a Convolutional Block Attention Module with residual blocks (detailed in Appendix 7). The model was pre-trained on the ImageNet dataset. After completion of pre-training, it was further trained on the target training set. During training, we used Adaptive Moment Estimation optimizer with the learning rate set at 1 × 10^−3^ without decay. The batch size was set to 64. The training process reached convergence around the 30th epoch, as illustrated by the loss curve provided in Supplementary Fig. S3. The prediction scores from the 2.5D-CNN were input to the Cox regression analysis to construct the DL model.

### Clinical model and combined model development

The clinicoradiological characteristics listed in Table 1 were employed to develop the clinical model. Table 2 shows the risk factors as determined by univariate and multivariate logistic regression analyses. By comparing the predictive efficacy and generalizability of the clinical, C-radiomics, ITH, and DL models, the appropriate models were selected to construct the final combined model. To ensure fairness and validity in model evaluation, we consistently employed the RF classifier for DM prediction. The specific methodologies are outlined in the aforementioned sections.

**Table 1:** Patient clinical characteristics

Variable	Training cohort (*n* = 142)		Internal validation cohort (*n* = 31)		External validation cohort (*n* = 101)		*p-*value
	Non-DM (*n* = 92)	DM (*n* = 50)	Non-DM (*n* = 19)	DM (*n* = 12)	non-DM (*n* = 80)	DM (*n* = 21)	
Age (years, median [IQR])	58.0 [48.75, 64.0]	57.5 [47.0, 62.5]	62.0 [45.0, 70.0]	64.0 [45.5, 69.75]	56.0 [46.5, 66.0]	62.0 [49.5, 73.0]	0.542
Sex (*n*, %)							0.869
Male	47 (51.1)	25 (50.0)	8 (42.1)	6 (50.0)	41 (51.25)	9 (42.9)	
Female	45 (48.9)	25 (50.0)	11 (57.9)	6 (50.0)	39 (48.75)	12 (57.1)	
Clinical T stage (*n*, %)							0.003
T1	8 (8.7)	2 (4.0)	1 (5.3)	0 (0.0)	15 (18.75)	1 (4.8)	
T2	34 (37.0)	11 (22.0)	6 (31.6)	3 (25.0)	20 (25.0)	7 (33.3)	
T3	14 (15.2)	14 (28.0)	5 (26.3)	3 (25.0)	28 (35.0)	8 (38.1)	
T4	36 (39.1)	23 (46.0)	7 (36.8)	6 (50.0)	17 (21.25)	5 (23.8)	
Clinical N stage (*n*, %)							0.179
N0	81 (88.0)	39 (78.0)	16 (84.2)	9 (75.0)	74 (92.5)	18 (85.7)	
N1	11 (12.0)	11 (22.0)	3 (15.8)	3 (25.0)	6 (7.5)	3 (14.3)	
Number (*n*, %)							< 0.001
Solitary	60 (65.2)	34 (68.0)	16 (84.2)	9 (75.0)	72 (90.0)	18 (85.7)	
Multiple	32 (34.8)	16 (32.0)	3 (15.8)	3 (25.0)	8 (10.0)	3 (14.3)	
Tumor shape (*n*, %)							< 0.001
Round	21 (22.8)	9 (18.0)	6 (31.6)	1 (8.3)	37 (46.75)	3 (14.3)	
Lobulated	21 (22.8)	6 (12.0)	3 (15.8)	6 (50.0)	18 (22.5)	7 (33.3)	
Irregular	50 (54.3)	35 (70.0)	10 (52.6)	5 (41.7)	25 (31.25)	11 (52.4)	
Cystic spaces or necrosis (*n*, %)							0.020
Not present	51 (55.4)	12 (24.0)	9 (47.4)	2 (16.7)	54 (67.5)	6 (28.6)	
Present	41 (44.6)	38 (76.0)	10 (52.6)	10 (83.3)	26 (32.5)	15 (71.4)	
Adipose tissue (*n*, %)							0.897
Not present	45 (48.9)	27 (54.0)	8 (42.1)	9 (75.0)	40 (50.0)	13 (61.9)	
Present	47 (51.1)	23 (46.0)	11 (57.9)	3 (25.0)	40 (50.0)	8 (38.1)	
Calcification (*n*, %)							0.485
Not present	80 (87.0)	41 (82.0)	14 (73.7)	10 (83.3)	67 (83.75)	20 (95.2)	
Present	12 (13.0)	9 (18.0)	5 (26.3)	2 (16.7)	13 (16.25)	1 (4.8)	
Enhancement pattern (*n*, %)							< 0.001
Heterogeneous	85 (92.4)	49 (98.0)	17 (89.5)	12 (100.0)	56 (70.0)	19 (90.5)	
Homogeneous	7 (7.6)	1 (2.0)	2 (10.5)	0 (0.0)	24 (30.0)	2 (9.5)	
Degree of enhancement (*n*, %)							< 0.001
Mild	39 (42.4)	19 (38.0)	7 (36.8)	1 (8.3)	35 (43.75)	10 (47.6)	
Moderate	33 (35.9)	14 (28.0)	9 (47.4)	6 (50.0)	40 (50.0)	10 (47.6)	
Marked	20 (21.7)	17 (34.0)	3 (15.8)	5 (41.7)	5 (6.25)	1 (4.8)	
Adjacent tissue involvement (*n*, %)							0.028
Not present	48 (52.2)	21 (42.0)	15 (78.9)	7 (58.3)	50 (62.5)	12 (57.1)	
Present	44 (47.8)	29 (58.0)	4 (21.1)	5 (41.7)	30 (37.5)	9 (42.9)	
Type of adjacent tissue involvement (*n*, %)							
Colon	13 (14.13)	15 (30.0)	2 (10.5)	2 (16.7)	4 (5.0)	1 (4.8)	0.002
Small bowel	9 (9.8)	2 (4.0)	0 (0.0)	1 (8.3)	2 (2.5)	0 (0.0)	0.118
Ureter	9 (9.8)	7 (14.0)	1 (5.3)	1 (8.3)	8 (10.0)	0 (0.0)	0.665
Bladder	3 (3.3)	5 (9.6)	0 (0.0)	0 (0.0)	1 (1.3)	0 (0.0)	0.080
Kidney	7 (7.6)	5 (10.0)	1 (5.3)	2 (16.7)	13 (16.3)	3 (14.3)	0.198
Adrenal	4 (4.3)	3 (6.0)	1 (5.3)	2 (16.7)	9 (11.3)	2 (9.5)	0.179
Uterus	1 (1.1)	1 (2.0)	0 (0.0)	0 (0.0)	0 (0.0)	0 (0.0)	0.617
Prostate	2 (2.2)	2 (4.0)	0 (0.0)	0 (0.0)	0 (0.0)	0 (0.0)	0.238
Muscle	8 (8.7)	14 (28.0)	1 (5.3)	1 (8.3)	6 (7.5)	2 (9.5)	0.152
Bone	0 (0.0)	1 (2.0)	0 (0.0)	1 (8.3)	1 (1.3)	1 (4.8)	0.335
Vessel	14 (15.2)	4 (8.0)	1 (5.3)	1 (8.3)	11 (13.8)	5 (23.8)	0.425
Histology (*n*, %)							0.001
WDLPS	20	5	7	1	33	7	
DDLPS	32	32	6	5	17	6	
Others LPS	9	1	0	0	7	0	
LMS	31	12	6	6	22	9	

The clinical T stage and N stage were based on the 8th edition of the American Joint Committee on Cancer (AJCC) staging system

*DM* distant metastasis, *IQR* interquartile range, *WDLPS* well-differentiated liposarcoma, *DDLPS* dedifferentiated liposarcoma, *LMS* leiomyosarcoma

**Table 2:** Analysis of distant metastasis-free survival through univariate and multivariate logistic regression in patients with RPS

Variable	Univariate logistic analysis		Multivariate logistic analysis	
	OR (95% CI)	*p*-value	OR (95% CI)	*p*-value
Age	0.992 (0.968, 1.016)	0.509	-	-
Sex	0.957 (0.481, 1.907)	0.902	-	-
Clinical T stage	1.374 (0.963, 1.961)	0.080	1.411 (0.973, 2.044)	0.069
Clinical N stage	2.077 (0.829, 5.206)	0.119	-	-
Number	0.882 (0.424, 1.836)	0.738	-	-
Tumor shape	0.889 (0.591, 1.338)	0.573	-	-
Cystic spaces or necrosis	3.074 (1.463, 6.458)	0.003	3.168 (1.491, 6.732)	0.003
Enhancement pattern	1.293 (0.842, 1.984)	0.240	-	-
Degree of enhancement	0.248 (0.030, 2.074)	0.198	-	-
Adipose tissue	0.816 (0.409, 1.626)	0.563	-	-
Calcification	1.463 (0.570, 3.756)	0.429	-	-
Adjacent tissue involvement	1.298 (0.570, 2.973)	0.248	-	-

*RPS* retroperitoneal sarcomas, *OR* odds ratio, *CI* confidence interval

### Statistical analysis

Statistical analyses were conducted using Python (version 3.9.7) and SPSS software (version 26.0). The normality of continuous variables was assessed using the Shapiro–Wilk test. Continuous variables were assessed for significance using either the t-test or the Mann–Whitney *U*-test, depending on their distribution. Categorical variables were compared using the Chi-square test or Fisher’s exact test. The performance of the models was evaluated using multiple metrics, including Harrell’s concordance index (C-index), the integrated Brier score (IBS), and the area under the curve (AUC). The fit of the models, their clinical reliability, and practical applicability were evaluated through calibration curves and decision curve analysis (DCA). Class activation maps (CAMs) were employed to track the regions of the RPS that the network identified as critical for distinguishing between DM and non-DM decisions. Patients were categorized into high-risk and low-risk groups based on the optimal cutoff values determined by X-tile software (Yale University School of Medicine, http://x-tile.software. informer.com). The Kaplan–Meier method, along with log-rank tests, was employed to estimate the DMFS for patients in two different risk groups. Statistical significance was established at *p*-values less than 0.05.

## Results

### Baseline information

A total of 274 patients were finally enrolled from two centers, including 142 patients in the training cohort, 31 patients in the internal validation cohort, and 101 patients in the external validation cohort. Among these, 86 (31.4%) patients had histologically confirmed RLMS, and 188 (68.6%) patients had histologically confirmed RLPS. DM occurred in 83 patients. Median DMFS was 15.5 months (range, 1–86 months) in the training cohort, 20.5 months (range, 3–90 months) in the internal validation cohort, and 33.2 months (range, 1–128 months) in the external validation cohort. The ICC for semantic features demonstrated a high level of consistency among the radiologists, as detailed in Table S3.

### Assessment of the efficacy of predictive models across internal and validation cohorts

**Fig. 2:**
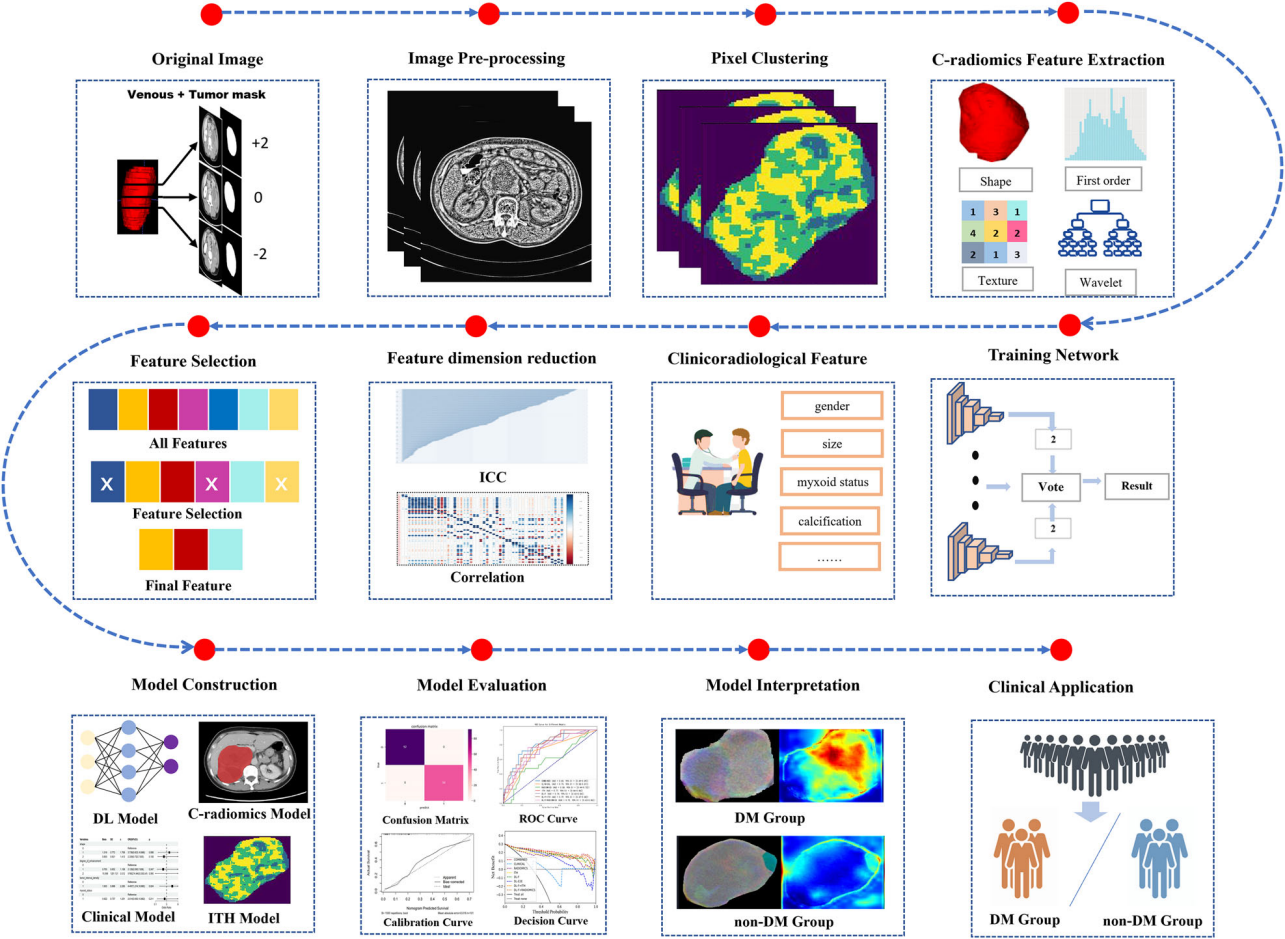
Workflow of the study. First, the original images were pre-processed, and unsupervised clustering was performed using manually delineated tumor regions. Second, ITH features, radiomic features, DL features, and clinicopathologic data were generated for each patient. Third, feature-selection processes were performed to select the features with predictive power. Finally, a combined model was constructed using the ITH-score, DL-score, and clinicopathologic data, and this model was assessed using validation cohorts. ITH, intratumoral heterogeneity; DL, deep learning

Figure 2 illustrates the research design. For predicting DM, the ITH model demonstrated satisfactory performance in both internal (AUC: 0.735; C-index: 0.691) and external (AUC: 0.765; C-index: 0.729) validation cohorts, slightly outperforming the clinical model (AUC: 0.695; C-index: 0.668) and C-radiomics (AUC: 0.581; C-index: 0.595) in the external validation cohort (Table 3). By integrating the ITH-score with the clinical, C-radiomics, and DL_F models, we constructed three new integrated models. The ablation study findings demonstrate that the ensemble models outperform their component models in validation datasets (Table S4).

**Table 3:** Model performances in the internal and external validation cohorts

Model	Internal validation cohort				External validation cohort		
	AUC		C-index	IBS	AUC	C-index	IBS
Clinical	0.654		0.585	0.133	0.695	0.668	0.043
C-radiomics	0.794		0.691	0.095	0.581	0.595	0.043
ITH	0.735		0.691	0.122	0.765	0.729	0.042
DL_F	0.873		0.786	0.117	0.741	0.725	0.043
DL_E	0.882		0.795	0.131	0.761	0.736	0.426
Combined	0.864		0.770	0.119	0.801	0.752	0.042

DeLong test	Internal validation cohort				External validation cohort		
	Difference between areas	Standard error		*p*-value	Difference between areas	Standard error	*p*-value
Combined vs. Clinical	0.213	0.083		0.010*	0.101	0.069	0.163
Combined vs. C-radiomics	0.068	0.083		0.159	0.219	0.075	0.004*
Combined vs. ITH	0.136	0.135		0.517	0.033	0.085	0.712
DL_F vs. Clinical	0.223	0.081		0.005*	0.043	0.079	0.569
DL_F vs. C-radiomics	0.078	0.071		0.265	0.161	0.079	0.045*
DL_F vs. ITH	0.146	0.136		0.310	0.025	0.090	0.785
DL_E vs. Clinical	0.232	0.083		0.005*	0.064	0.074	0.378
DL_E vs. C-radiomics	0.087	0.070		0.208	0.181	0.080	0.024*
DL_E vs. ITH	0.154	0.134		0.257	0.004	0.087	0.966

*p < 0.05

The AUCs among models were compared using the DeLong test

*ITH* intratumoral heterogeneity, *DL_F* deep-learning feature extraction, *DL_E* end-to-end deep learning, *AUC* area under the receiver operating characteristic curve

The SHAP summary plot (Fig. S4, left panel) systematically quantifies the relative contributions of clinicoradiological variables in predicting the distant metastasis of RPS. Among these variables, cystic spaces or necrosis emerged as the most significant imaging biomarker (mean |SHAP value | = 0.573), followed by clinical T-stage, which ranked second in predictive importance (mean |SHAP value | = 0.463). The SHAP value distribution plot (Fig. S4, right panel) visualizes how these clinicoradiological variables influence the model’s output. Each dot represents an individual sample, with the *x*-axis showing the SHAP value (the feature’s contribution to the model’s prediction) and the *y*-axis listing the features ranked by importance. The color gradient (ranging from purple to yellow) indicates the feature value, with purple representing lower values and yellow representing higher values. Among these, cystic spaces or necrosis show a strong correlation with changes in the model output, with higher feature values generally associated with higher SHAP values.

The combined model integrating clinicoradiological variables with the ITH-score and DL-score demonstrated the best predictive performance. Specifically, the combined model achieved an AUC of 0.864 and a C-index of 0.770 in the internal validation cohort, and an AUC of 0.801 and C-index of 0.752 in the external validation cohort. The combined model exhibited an IBS value of 0.119 in the internal validation cohort and 0.042 in the external validation cohort, indicating a reduced likelihood of prediction errors. In the internal validation cohort, the AUCs of the combined model, DL_F model, and DL_E model significantly surpassed the clinical model. Similarly, in the external validation cohort, the AUCs of these three models significantly exceeded that of the C-radiomics model. Figure S5 shows the CAM heatmaps for patients with different DM states.

**Fig. 3:**
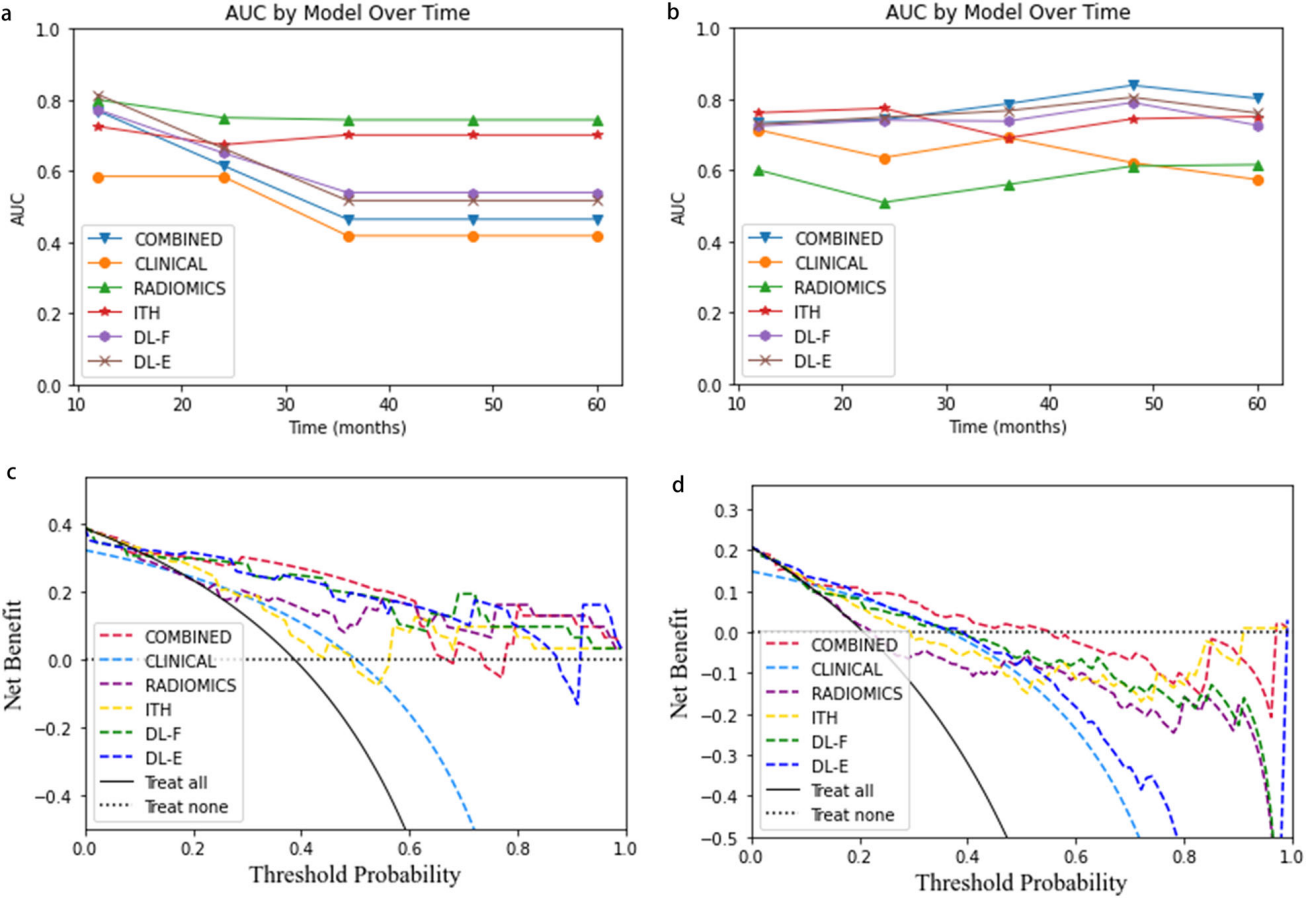
Predictive efficacy of the six developed models for DMFS. **a**, **b** represent time-dependent ROC curves for the internal and the external validation cohorts. **c**, **d** represent the corresponding DCA plots in the internal and validation cohorts. DMFS, distant metastasis-free survival; ROC, receiver operating characteristic; DCA, decision curve analysis

**Fig. 4:**
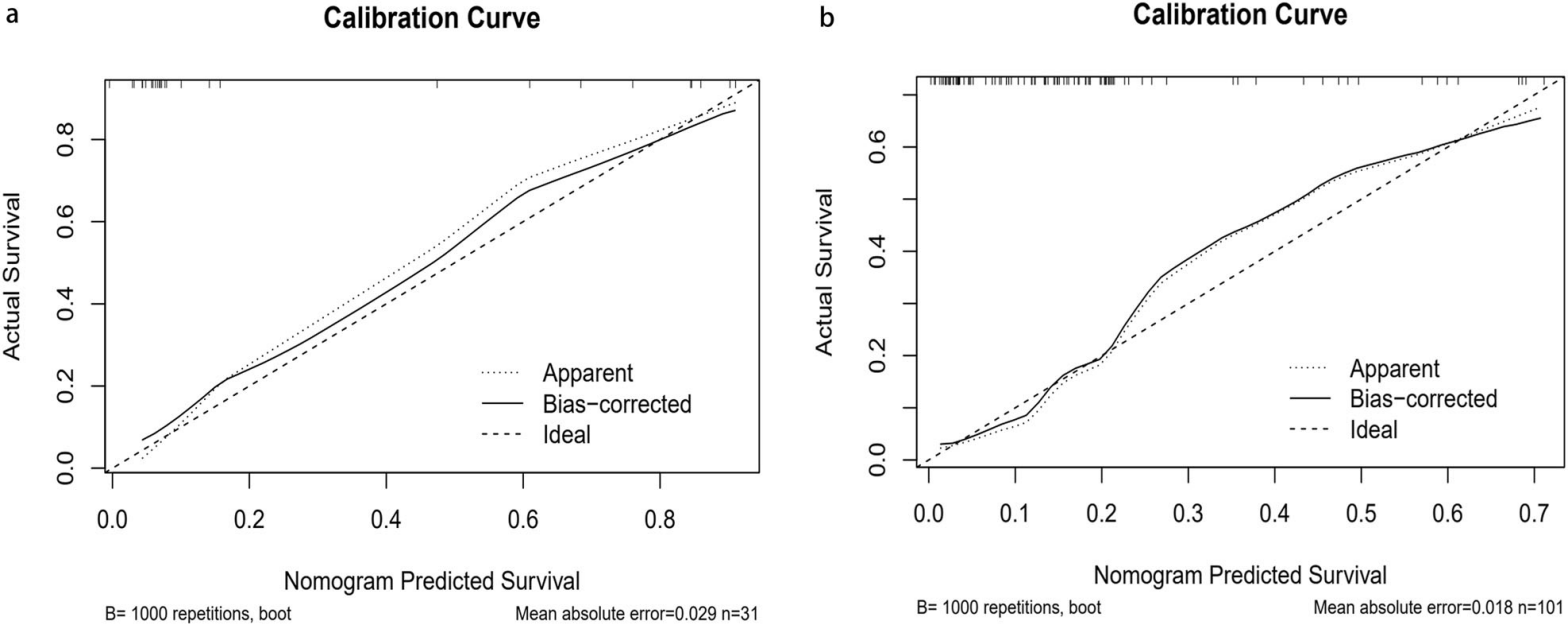
Evaluation of the combined model in the multicenter cohorts. **a**, **b** represent the calibration plots in the internal and external validation cohorts

**Fig. 5:**
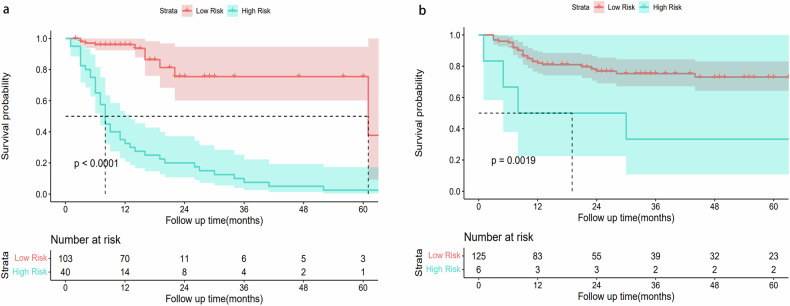
Kaplan–Meier curves based on the combined model for predicting DMFS in RPS. **a**, **b** represent the comparison between the high-risk and low-risk groups from both the training and validation sets. DMFS, distant metastasis-free survival; RPS, retroperitoneal sarcomas

In the analysis based on time-dependent ROC (Fig. 3a, b), the AUCs of the combined model were higher than those of the clinical model, C-radiomics, and DL_F model at different time points in the external validation set. Detailed information regarding the AUCs can be found in Table S5. Using net benefit as the DCA evaluation metric, the combined model demonstrated superior clinical net benefit compared to the other models. This was evident in the external validation cohort, with clinical thresholds set between 0.2 and 0.9 (Fig. 3c, d). Calibration curves for the combined model’s predictions of DMFS demonstrated a reasonable concordance between the predicted probabilities and actual DM occurrence in the internal and external validation cohorts (Fig. 4a, b). Figure 5a, b present the Kaplan–Meier curves for DMFS in both the training and validation sets. These curves indicate that the combined model significantly stratifies patients by DMFS in all datasets (*p* < 0.05 for each, log-rank test). Figure S6 illustrates two patients at the same clinical stage with comparable clinicoradiological features but markedly different prognoses. Patient A was predicted by the model to be at high risk for DM and experienced metastasis after 8 months, whereas Patient B, classified as showing low risk for DM, did not exhibit metastasis by the end of the follow-up period.

### Evaluation of model performance in patient subgroups

**Fig. 6:**
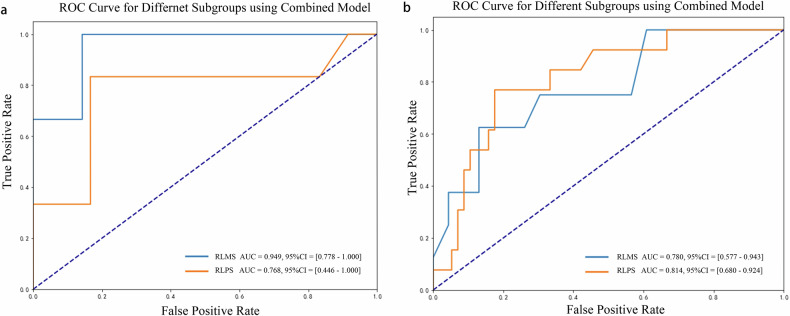
Performance of the combined model across various histological subtypes of RPS. **a**, **b** represent the ROC curves for RLMS and RLPS in the internal and external validation cohorts. No significant difference in AUC is observed for different types of RPS within the internal (*p* = 0.838) and external (*p* = 0.220) cohorts. RPS, retroperitoneal sarcomas; ROC, receiver operating characteristic; RLMS, retroperitoneal leiomyosarcoma; RLPS, retroperitoneal liposarcoma; AUC, area under the receiver operating characteristic curve

Since over 65% of patients in this study had RLPS, we examined the potential influence of tumor types on the evaluation performance of the model. As illustrated in Fig. 6, the AUCs of the combined model for RLMS and RLPS reached 0.952 and 0.771 in the internal validation cohort, and 0.777 and 0.814 in the external validation cohort. No statistically significant disparity was found in the diagnostic performance between RLMS and RLPS within the internal (*p* = 0.260) and external (*p* = 0.754) validation groups.

## Discussion

Accurate DM prediction is very beneficial for selecting appropriate treatment and stratifying risks for RPS patients. Our research validated the predictive power of C-radiomics, ITH, DL_F, and DL_E models based on the CECT images, alongside a clinical model, for forecasting DMFS in patients with RPS undergoing surgical therapy. The ITH model using intratumoral ecological diversity features proved to be reliable for DM prediction. A model combining the ITH-score, DL-score, and clinicoradiological variables showed superior performance, achieving excellent predictive power for both DMFS prediction and risk stratification. This combined model achieved a C-index of 0.752 and an AUC of 0.801 in validation sets, with a smaller prediction error (IBS value of 0.042), demonstrating its significance in prognostic assessment.

The use of radiomics and DL algorithms to predict DM in patients with RPS is a promising approach. However, the clinical application of radiomics and DL techniques is hindered by challenges such as medical imaging data heterogeneity and limited generalizability across multicentric cohorts, underscoring the need for innovative methodologies to overcome these barriers and ensure the effective implementation of these models in clinical practice [33, 34]. Tian et al [18] developed DL and radiomics models for DM prediction in RLMS by using CECT images, achieving AUCs ranging from 0.733 to 0.786 in the validation group. However, this study was limited to RLMS, had a small sample size, and did not consider the influence of tumor heterogeneity. Our study incorporated cases of both RLPS and RLMS into predictive models to enhance their clinical utility. RLPS and RLMS constitute the primary histological subtypes of RPS, accounting for 50%–63% and 19%–23% of the cases, respectively [3, 4]. The C-radiomics model showed robust performance in the internal validation set but was less effective in the external set, indicating potential generalizability issues likely influenced by data heterogeneity from diverse centers and scanners. This highlights the real-world data heterogeneity challenge in model development. To address issues of generalizability, our research employed the following experimental design strategy:

Initially, we designed a residual network adapted for 2.5D medical imaging that incorporated both channel and spatial attention mechanisms. This process enhanced feature selection, markedly improving the model’s precision and efficiency for image processing. The DL_F and DL_E models demonstrated better performance on the training set (AUC: 0.903–0.908) compared to the internal validation set (AUC: 0.873–0.882) and the external validation set (AUC: 0.741–0.761), indicating a degree of overfitting. Despite attempts to enhance generalizability, the complexity of DL models necessitates additional strategies to mitigate overfitting and improve generalizability. Subsequently, we employed CECT features predictive of DM, utilizing both univariate and multivariate analyses, to establish a clinical model. The results indicate that the clinical model was less effective than C-radiomics in the internal validation but showed a slight advantage over it in external validation. The enhanced external validation performance of the clinical model may be attributable to its insulation from scanner-specific and parameter-related variability, which is a limitation of the C-radiomics model. To minimize inter-reader variability, three abdominal radiologists independently assessed the features, achieving ICC values of over 0.900 for agreement. Thus, the use of clinical features is a potent regularization technique to bolster model generalizability and reduce overfitting. Although combining clinical and radiomics models modestly improved generalizability (internal: 0.879; external: 0.678), it did not remove overfitting. Therefore, we introduced the ITH-score as a novel method.

Despite the recognized correlation between ITH and the risk of DM [22, 24, 35], previous quantitative imaging analyses have not comprehensively evaluated the connections among C-radiomics features, ITH, and DMFS. Consequently, quantifying tumor heterogeneity accurately and incorporating this information effectively into routine clinical practice remains a challenge. Our study employed a radiomics method to introduce straightforward and noninvasive intratumoral ecological diversity features that precisely reflect the ITH of RLPS and RLMS. The findings revealed that the ITH model achieved commendable performance on both the internal (AUC: 0.735) and the external (AUC: 0.765) validation sets. Furthermore, ablation analysis of the ITH model (refer to Table S4) highlighted the model’s strong capacity for informational representation and its versatility across different validation cohorts. Thus, the ITH model surpassed the C-radiomics model in generalizability while maintaining robust informational capacity. This could assist clinicians in better selecting adjuvant therapies by identifying patients who would benefit from more aggressive treatments and avoiding unnecessary therapies for low-risk patients, all through more reliable predictions across diverse patient cohorts.

Given the robust generalizability of both the clinical and ITH models, we integrated them with DL techniques as a form of regularization. To achieve this, we employed a DL model for feature extraction from the data, which was then combined with clinical features and the ITH-score. The findings indicated that the combined model significantly enhanced performance in the external validation set. This demonstrated the enhanced generalizability of the combined model, validating the robust generalization capabilities of the ITH and clinical features, as well as their effectiveness as regularization terms. Additionally, subgroup analysis revealed that the combined model exhibited strong predictive performance for both RLMS and RLPS, further supporting its utility in these contexts.

Although our study yielded encouraging outcomes, its inherent limitations require consideration. First, the study was a retrospective analysis and was consequently subject to selection bias and inherent biases. Although external validation was conducted to enhance reliability, the rarity of RPS necessitated the use of retrospective cohorts. Nonetheless, the valuable findings from this study should be further consolidated and validated through prospective studies. Second, given the rarity of other retroperitoneal pathologies, our study was necessarily limited to RLPS and RLMS, which are the most prevalent histological subtypes of RPS and constitute approximately 50%–63% and 19%–23% of the cases of RPS, respectively [3, 4]. Consequently, the generalizability of our model to all subtypes of RPSs may be limited. Additionally, the small sample size, due to the low incidence of RPS, underscores the need for validation with larger, multicenter datasets. Nonetheless, this study surpasses most prior radiomics analyses of this tumor type and anatomical site. Finally, manual tumor delineation requires expertise and is susceptible to subjective biases. Although we have endeavored to mitigate this issue by selecting features with ICC values exceeding 0.90, an automated and precise tumor-segmentation approach is crucial to guarantee the stability and reproducibility and enhance the efficiency of imaging analysis in the process.

In conclusion, the ITH model using intratumoral ecological diversity features proved to be reliable for DM prediction. Additionally, a combined model incorporating the ITH-score, DL-score, and clinicoradiological variables was an effective tool for DM risk stratification of RLPS and RLMS, potentially facilitating clinical decision-making in precision medicine.

## Supplementary information

ELECTRONIC SUPPLEMENTARY MATERIAL

## Data Availability

The datasets generated during and/or analyzed during the current study are available from the corresponding author on reasonable request.

## Abbreviations

AUC: Area under the receiver operating characteristic curve
CAM: Class activation maps
CECT: Contrast-enhanced computed tomography
DCA: Decision curve analysis
DL: Deep learning
DMFS: Distant metastasis-free survival
ICC: Intraclass correlation coefficient
ITH: Intratumoral heterogeneity
RLMS: Retroperitoneal leiomyosarcoma
RLPS: Retroperitoneal liposarcoma
RPS: Retroperitoneal sarcoma
