# Supplementary material for: CT-based quantification of intratumoral heterogeneity for predicting distant metastasis in retroperitoneal sarcoma
Source: Insights Imaging. 2025 May 9;16:99. doi: 10.1186/s13244-025-01977-9 (PMC12064543; doi:10.1186/s13244-025-01977-9)
Supplement: Supplementary file 1 — ELECTRONIC SUPPLEMENTARY MATERIAL [file 13244_2025_1977_MOESM1_ESM.pdf]

CT-based Quantification of Intratumoral Heterogeneity for Predicting Distant Metastasis in Retroperitoneal Sarcoma

ELECTRONIC SUPPLEMENTARY MATERIAL

Table S1. Settings used for CT scans at the two institutions

Parameters	Institution A	Institution B
CT version	Revolution CT or LightSpeed CT750 HD (all from GE Healthcare); SOMATOM Sensation 64, or Force or Definition Flash (all from Siemens)	uCT960 or uCT710 (all from United Imaging); Revolution CT, or LightSpeed CT750 HD, 64 LightSpeed VCT (all from GE Healthcare); Definition Flash (Siemens)
Tube current	Automatic tube current modulation	Automatic tube current modulation
Tube voltage	120kV	100 kV
Detector collimation	0.625mm or 0.6mm	0.625mm or 0.6mm
Slice thickness	5mm	5mm
Pitch	1.0 or 1.375	0.6 or 0.8
Image matrix	512×512	512×512
Contrast material	Iopromide 370 (Bayer)	Iopamidol 350 (Hengrui)

Abbreviations: CT, computed tomography

Table S2 The results for cluster numbers ranging from 2 to 8 in the internal and external validation cohorts

**Cluster numbers**	**Internal validation cohort**		**External validation cohort**	
	**Accuracy**	**AUC**	**Accuracy**	**AUC**
**2**	0.645	0.708	0.554	0.669
**3**	0.677	0.640	0.574	0.560
**4**	0.710	0.726	0.525	0.550
**5**	0.581	0.693	0.624	0.615
**6**	0.548	0.548	0.515	0.507
**7**	0.419	0.423	0.465	0.466
**8**	0.645	0.675	0.525	0.467
**2-5**	0.742	0.735	0.703	0.765

Abbreviations: AUC, area under the receiver operating characteristic curve

Table S3. Interobserver agreement of CECT semantic features for retroperitoneal tumors

CECT semantic feature	ICC	95% CI
Clinical T stage	0.984	0.981 - 0.987
Clinical N stage	0.959	0.950 – 0.967
Number	1.000	1.000 - 1.000
Tumor shape	0.971	0.965 - 0.977
Cystic spaces or necrosis	0.947	0.935 - 0.956
Enhancement pattern	0.850	0.820 - 0.876
Degree of enhancement	0.931	0.917 - 0.944
Adipose tissue	0.976	0.970 - 0.980
Calcification	0.964	0.956 – 0.970
Adjacent organ involvement	0.990	0.988 – 0.992

Abbreviations: CECT, contrast-enhanced computed tomography; ICC, intraclass correlation coefficient;
CI, confidence interval.

ICC values of 0–0·20 indicate poor agreement; 0·21–0·40 indicates fair agreement; 0·41–0·60 indicates moderate agreement; 0·61–0·80 indicates good agreement; and >0·80 indicates excellent agreement.

Table S4. The ablation and comparative experiment to validate model performance

Model	Internal validation cohort			External validation cohort		
	C-index	AUC	IBS	C-index	AUC	IBS
**Clinical**	0.585	0.654	0.133	0.668	0.695	0.043
**C-radiomics**	0.691	0.794	0.095	0.595	0.581	0.043
**DL_F**	0.786	0.873	0.117	0.725	0.741	0.043
**Clinical + ITH**	0.756	0.750	0.123	0.691	0.790	0.041
**C-radiomics + ITH**	0.616	0.846	0.137	0.712	0.632	0.044
**DL_F + ITH**	0.799	0.879	0.115	0.750	0.784	0.041

Abbreviations: ITH, intratumoral heterogeneity, DL_F, deep learning feature extraction; C-index; Harrell's concordance index; AUC, area under the receiver operating characteristic curve; IBS, integrated Brier score.

Table S5. Performance of various machine learning classifiers with reference to the deep learning radiomics signature

Types	Time (month)	Internal validation cohort		External validation cohort	
		AUC	95% CI	AUC	95% CI
**Clinical model**	12	0.585	0.472, 0.707	0.713	0.628, 0.804
	24	0.585	0.393, 0.750	0.635	0.599, 0.774
	36	0.418	0.336, 0.500	0.692	0.623, 0.802
	48	-	-	0.621	0.555, 0.760
	60	-	-	0.574	0.503, 0.728
**C-radiomics model**	12	0.800	0.639, 0.939	0.601	0.485, 0.747
	24	0.750	0.559, 0.948	0.510	0.467, 0.676
	36	0.744	0.506, 1.000	0.560	0.486, 0.707
	48	-	-	0.612	0.513, 0.747
	60	-	-	0.616	0.495, 0.755
**ITH model**	12	0.725	0.602, 0.895	0.762	0.674, 0.868
	24	0.674	0.543, 0.904	0.774	0.694, 0.871
	36	0.701	0.524, 1.000	0.691	0.635, 0.832
	48	-	-	0.745	0.678, 0.866
	60	-	-	0.751	0.676, 0.896
**DL_F model**	12	0.772	0.637, 0.937	0.725	0.633, 0.841
	24	0.651	0.437, 0.901	0.741	0.643, 0.831
	36	0.539	0.314, 0.874	0.738	0.627, 0.824
	48	-	-	0.791	0.676, 0.865
	60	-	-	0.727	0.603, 0.828
**DL_E model**	12	0.815	0.679, 0.939	0.729	0.653, 0.842
	24	0.664	0.440, 0.874	0.749	0.664, 0.841
	36	0.517	0.314, 0.782	0.768	0.653, 0.847
	48	-	-	0.805	0.692, 0.873
	60	-	-	0.761	0.636, 0.853
**Combined model**	12	0.769	0.622, 0.886	0.735	0.643, 0.848
	24	0.616	0.363, 0.812	0.742	0.667, 0.841
	36	0.464	0.178, 0.745	0.787	0.699. 0.865
	48	-	-	0.839	0.738, 0.908
	60	-	-	0.802	0.694, 0.899

Abbreviations: AUC, area under the receiver operating characteristic curve; CI, confidence interval; ITH, intratumoral heterogeneity; DL_F, deep learning feature extraction; DL_E, end to end deep learning; AUC, area under the receiver operating characteristic curve.

Appendix 1:

The inclusion criteria were as follows: (1) Histologically diagnosed with RLMS or RLPS; (2) underwent surgical resection and achieved a margin-negative (R0) status; (3) Completion of the preoperative non-contrast-enhanced CT and contrast-enhanced CT < 15 days before treatment; (4) Favorable images quality for analysis (5) Accessibility of complete clinical and pathological data**.**

The main exclusion criteria were as follows: (1) Deficient clinical and pathological indicator or imaging information; (2) Inadequate imaging quality; (3) Previous malignancy or other coexisting malignant tumors; (4) Treatment with anticancer therapy at or before the baseline CECT scans were conducted; (5) Death from non-metastatic causes within the 6 months following surgery.

Appendix 2:

The details of the semantic features included are as follows:(1) tumor number, (2) shape, (3) presence of cystic spaces or necrosis (necrosis or cystic components, 0-20 Hounsfield units [HU]), (4) enhancement pattern (homogeneous or heterogeneous, where heterogeneous was recorded when heterogeneity was present in >30% of the entire mass volume), (5) degree of enhancement (mild enhancement, an increase of <20 HU; moderate enhancement, an increase of 20–40 HU; and marked enhancement, an increase of >40 HU, compared to plain CT values), (6) adipose tissue, (7) calcification, (8) adjacent tissue involvement, and (9) the type of adjacent tissue involvement.

Appendix 3:

Manual segmentation of tumor region was performed on venous-phase images by experienced radiologists. The three-dimensional tumor region was manually delineated by two experienced radiologists (X.X. and X.X., with 6 and 8 years of experience in abdominal imaging diagnosis) on venous-phase images using the ITK-SNAP software (version 3·8·0, available at www.itksnap.org). They carefully avoided normal tissue to ensure accurate delineation of the entire tumor. Three weeks later, 60 cases were randomly selected from the entire cohort and independently segmented to calculate the inter- and intra-observer re-producibilities (ICCs).

Appendix 4:

The venous-phase images underwent adaptive histogram equalization to enhance contrast while preserving local details. Normalization scales the pixel values from the range of [0, 255] to [0, 1], thus speeding up convergence during model training, minimizing biases in data distribution, and ultimately enhancing the generalization capabilities of the model. To preserve lesion margins, a morphological dilation (using a 4 × 4 kernel) was applied to the ROI. The image was then cropped based on the expanded ROI region, and all pixels outside the ROI were set to 0, optimizing computational efficiency and minimizing noise. The slice containing the largest tumor area was designated as the nth slice. Subsequently, slices at the (n - 2)th, nth, and (n + 2)th positions were extracted and stacked along the channel dimension to construct a 2·5-dimensional (2·5D) DL model. All the stacked images were resized to 224 pixels × 224 pixels through bilinear interpolation. To avoid overfitting and boost model robustness, extensive data augmentation is crucial for DL models trained on limited datasets. We implemented comprehensive transformations, including both horizontal and vertical flips, as well as rotations spanning 0° to 360° in 45° increments (e.g., 45°, 90°, 135°, etc.), on all images in training set.

Appendix 5:

The ComBat compensation method is applied primarily to remove batch effects stemming from technical variability or scanner discrepancies across image batches. These effects can distort feature representation between batches, compromising result accuracy and reliability. ComBat adjusts the data to enhance comparability across batches and maintains sample biological variation, thus improving result interpretability.

The key benefits of the ComBat method are as follows:

1. Batch effect correction: ComBat effectively addresses technical and instrumental batch effects, ensuring data comparability.
2. Conservation of biological variation: It preserves sample biological differences during correction, retaining critical biological information.
3. Versatility: ComBat is a versatile tool applicable to diverse image genomics data, such as gene, protein, and metabolite expression, fitting various research designs and data types.

Appendix 6:

Modifying the count of target clusters in clustering yields varying ITH values. Table S2 outlines the model outcomes for cluster numbers ranging from 2 to 8. The results show that RF models using cluster numbers of 2, 3, 4, and 5 exhibited superior performance on both validation sets. To maintain model stability, we chose 2, 3, 4, and 5 clusters to derive ITH values, securing a robust ITH model. We employed the RF classifier to devise a predictive model, augmented it with the synthetic minority oversampling technique to mitigate the issue of imbalanced data, and leveraged Bayesian hyperparameter optimization along with ten-fold cross-validation to achieve automatic model tuning.

Appendix 7:

Convolutional Block Attention Module leverages both channel and spatial attention mechanisms to enhance feature learning by focusing on important features across the channel and spatial dimensions. By incorporating residual blocks, the network benefits from improved gradient flow, allowing easier training of deeper models effectively. The Mish activation function is used to boost convergence and robustness by providing smooth gradients, which helps avoid issues associated with sharp activation transitions. Additionally, dropout layers in the classifier are utilized to improve generalization and mitigate overfitting by randomly deactivating neurons during training, thereby preventing the model from becoming too reliant on any specific features. This combination of techniques ensures that our model not only performs well but also generalizes effectively to new data.

The model was trained, optimized, and evaluated using an Intel i7-12700H 14-core CPU and an RTX 4080 24 GB GPU, leveraging PyTorch (Version: 2·0·0, https://pytorch.org/) and CUDA (Version 11·6·0, Nvidia).

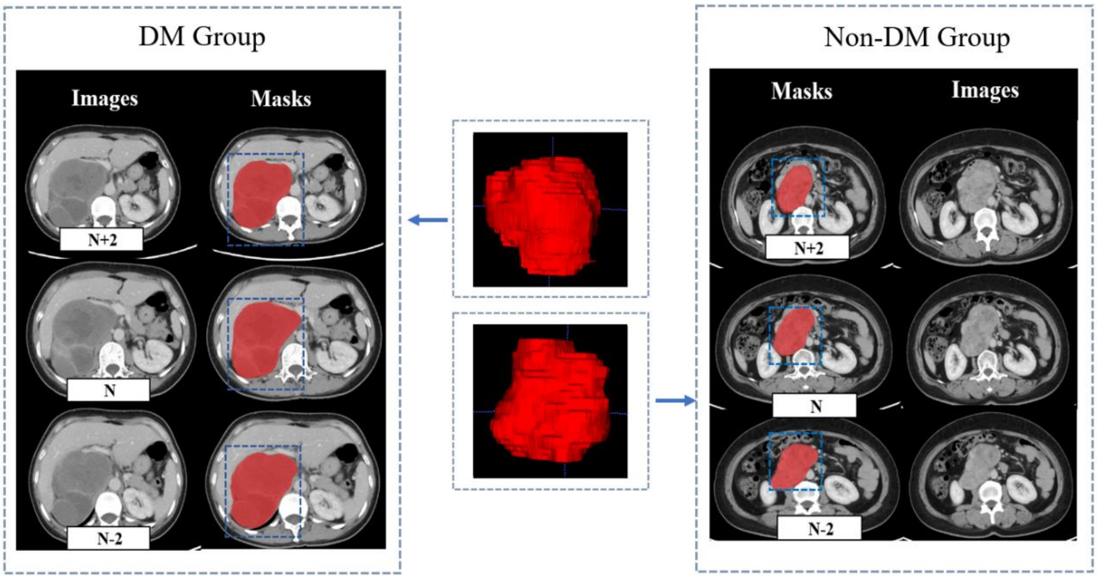

Figure S1. Three-dimensional delineation of the RLMS with DM and the RLMS without DM was performed manually on the venous phase of CECT images.

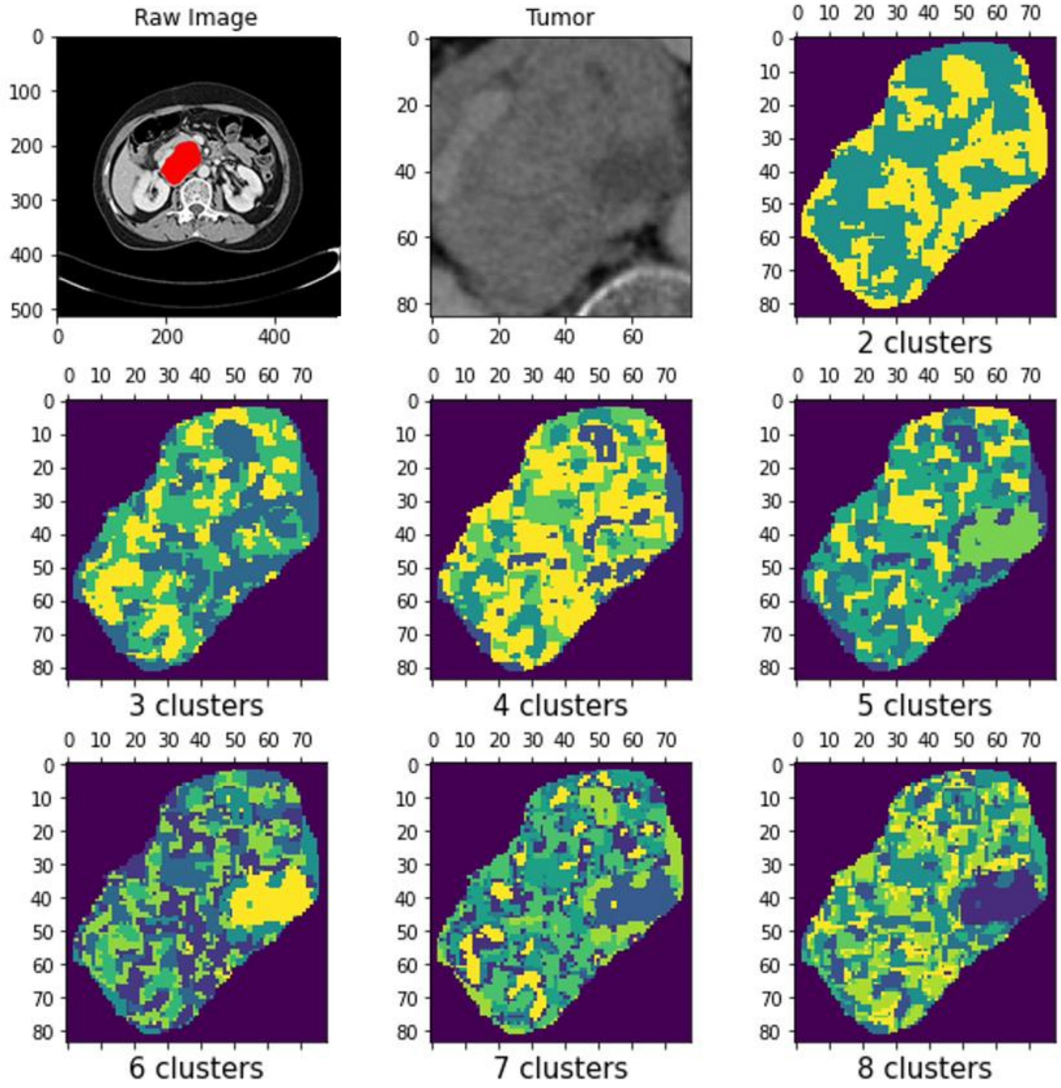

Figure S2. Illustration of unsupervised clustering

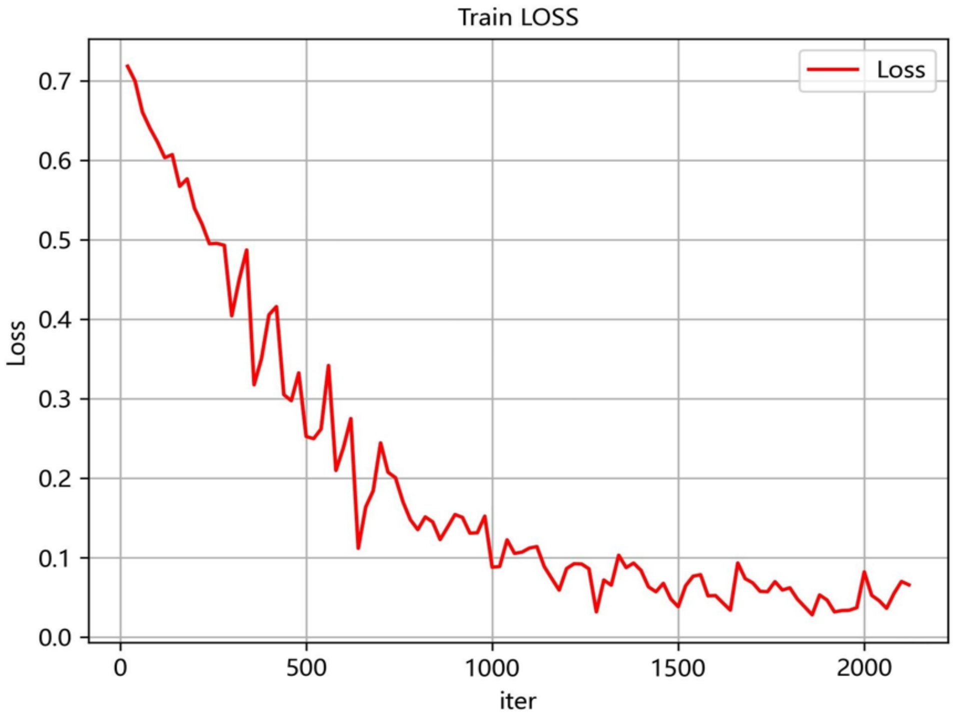

Figure S3. Training loss curve of the DL model**.** The figure shows that loss of the training cohort decreases and gradually converges with an increasing number of iterations.

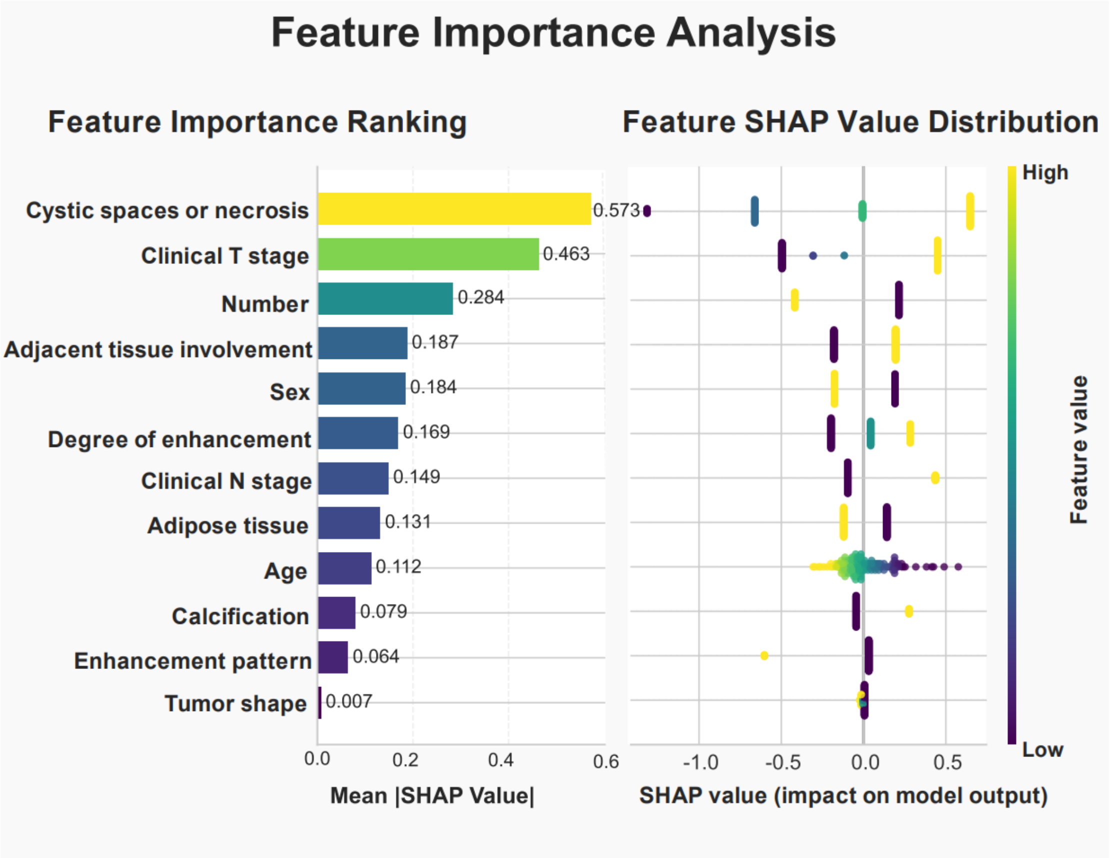

Figure S4. SHAP value analysis of clinicoradiological features in predicting distant metastasis of RPS. (Left panel) The SHAP summary plot quantifies the relative contributions of clinicoradiological features in predicting distant metastasis of RPS. (Right panel) The SHAP value distribution plot visualizes how clinicoradiological features influence the model output. Each dot represents an individual sample, with the x-axis showing the SHAP value (feature contribution) and the y-axis listing features ranked by importance. The color gradient (purple to yellow) indicates the feature value, with purple representing lower values and yellow representing higher values.

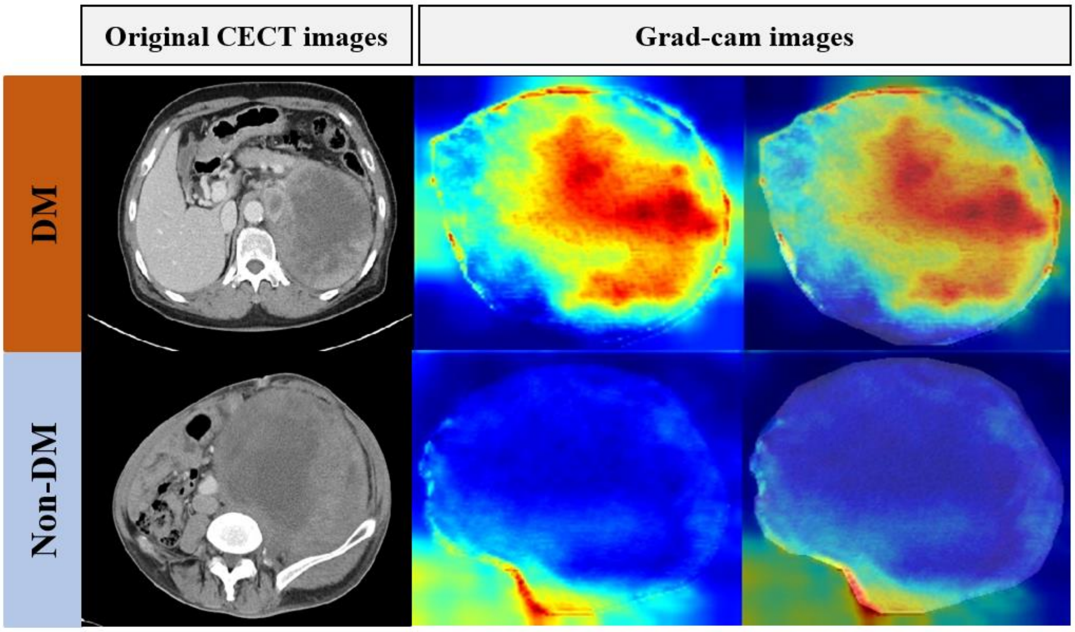

Figure S5. The activation maps illustrate the significant RPS regions that the convolutional neural networks have learned to be the most indicative of DM. The areas highlighted correspond to regions of high predictive value in the model's DM forecasts, while the less emphasized regions signify areas of minimal importance in predicting DM.

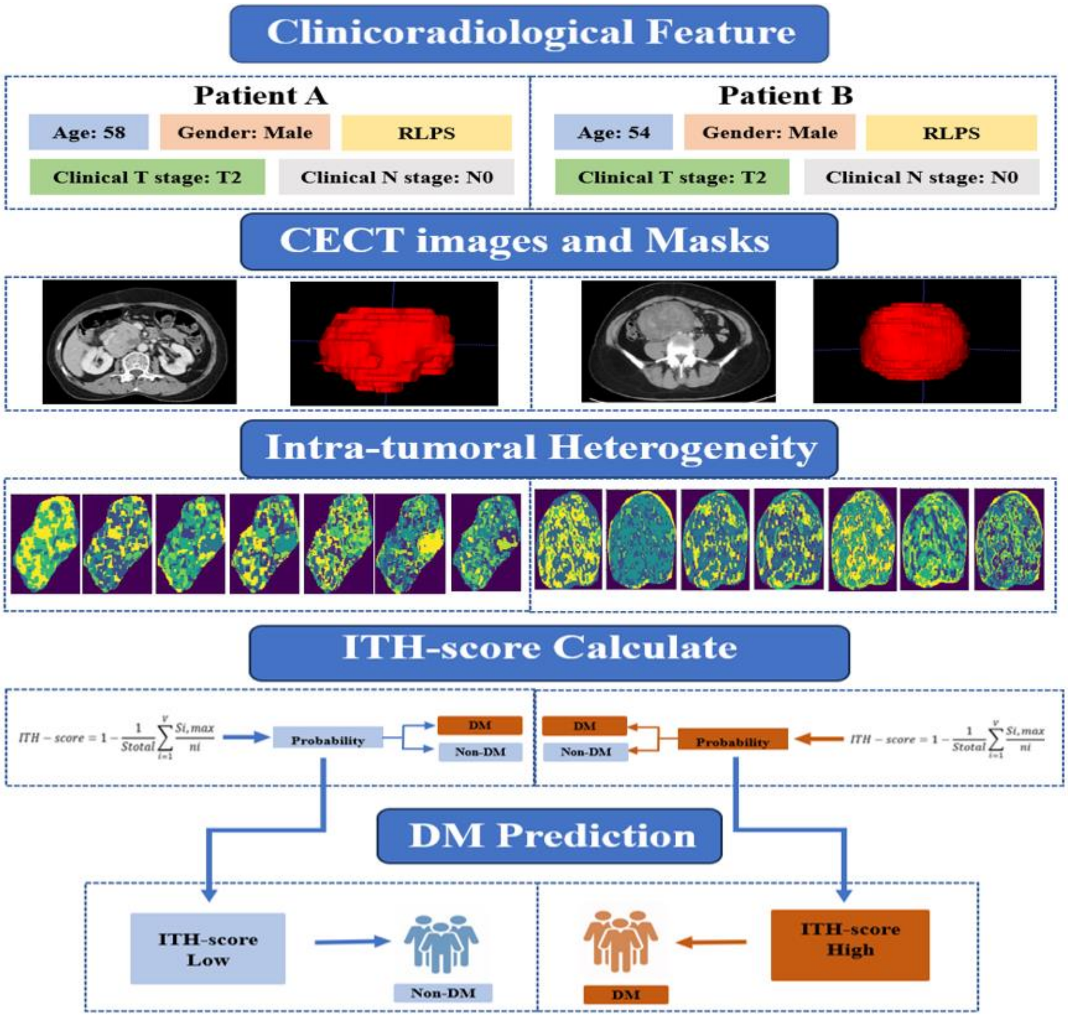

Figure S6. The schematic depicts the use of the intratumoral heterogeneity (ITH) score in two patients with retroperitoneal sarcomas who exhibited similar clinical traits. Both patients A and B presented with the same pathological type, sex, clinical T and N stages, and were of similar age (58 and 54 years, respectively). Computed tomography images delineating the tumor regions are displayed for both patients. We performed an analysis by varying the number of clusters from 2 to 8 and computed the ITH-score for each configuration. The ITH-score ranged from 0 to 1, with higher scores signifying greater heterogeneity in the label map, indicative of increased variability in cellular composition and spatial distribution within the tissue. Patient A, classified as low risk for distant metastasis (DM), did not develop metastasis by the end of the follow-up period. In contrast, Patient B was predicted by the model to be at high risk for DM and developed metastasis after 8 months.
